# Correction: Structural basis for plant plasma membrane protein dynamics and organization into functional nanodomains

**DOI:** 10.7554/eLife.66677

**Published:** 2021-01-27

**Authors:** Julien Gronnier, Jean-Marc Crowet, Birgit Habenstein, Mehmet Nail Nasir, Vincent Bayle, Eric Hosy, Matthieu Pierre Platre, Paul Bernard Gouguet, Sylvain Raffaele, Denis Martinez, Axelle Grelard, Antoine Loquet, Francoise Simon-Plas, Patricia Gerbeau-Pissot, Christophe Der, Emmanuelle M Bayer, Yvon Jaillais, Magali Deleu, Véronique Germain, Laurence Lins, Sébastien Mongrand

Gronnier J, Crowet J-M, Habenstein B, Nasir MN, Bayle V, Hosy E, Platre MP, Gouguet P, Raffaele S, Martinez D, Grelard A, Loquet A, Simon-Plas F, Gerbeau-Pissot P, Der C, Bayer EM, Jaillais Y, Deleu M, Germain V, Lins L, Mongrand S. 2017. Structural basis for plant plasma membrane protein dynamics and organization into functional nanodomains. *eLife* **6**:e26404. doi: 10.7554/eLife.26404.

Published 31, July 2017

Two errors were identified in Figure 1B right/1E left and in Figure 4-Supplement figure 2, where we accidentally duplicated panels. These errors occurred during figures assembly in Adobe Illustrator.

In addition, by scrutinizing for additional potential mistakes we realized that representative pictures of PVX-GFP infection foci presented in Figure 1-supplement 2 for Mock and RFP-StREM1.3 do not originate from this set of experiment. While these does not affect the appearance of the panel, we replaced these images for consistency. This error occurred during the preparation of the successive versions of this figure which originally included additional PVX-GFP infection experiments.

Importantly, these corrections do not affect neither the results nor the conclusions of the original paper. We apologize for the confusion this may have led during the reading of the manuscript.

Corrected Figure 1:

**Figure 1:**
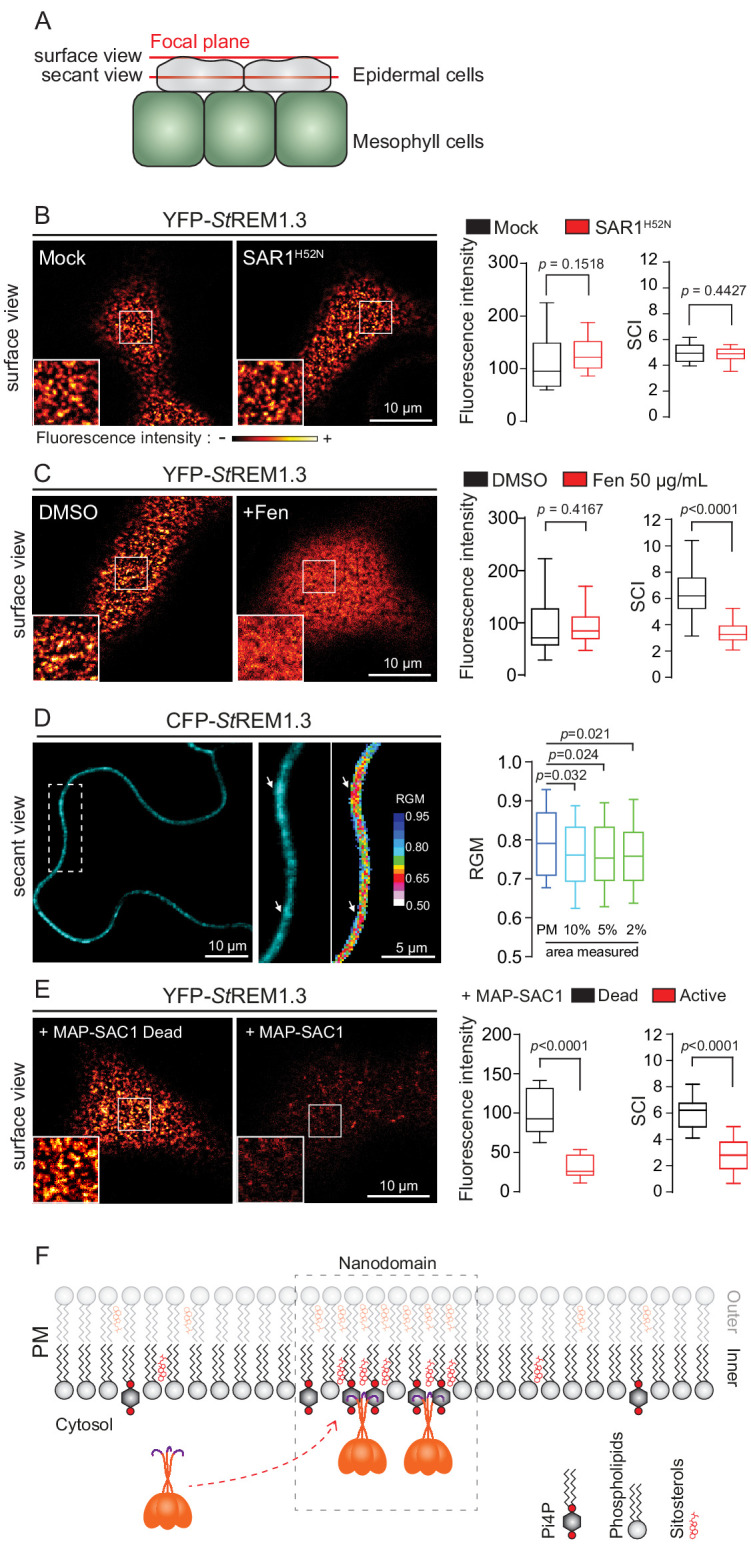


Original Figure 1:

**Figure 2:**
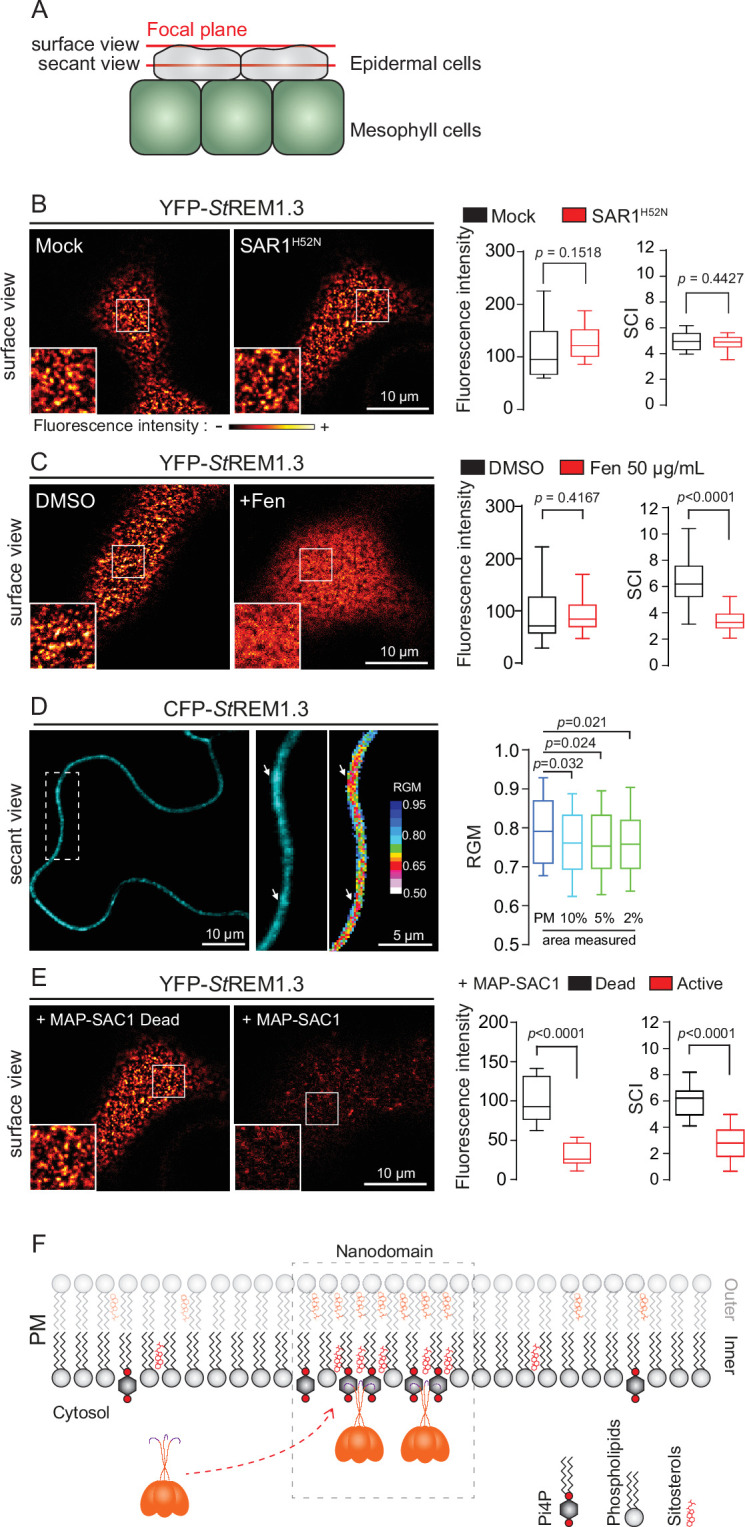


Corrected Figure 1 – figure supplement 2 panel G:

**Figure 3:**
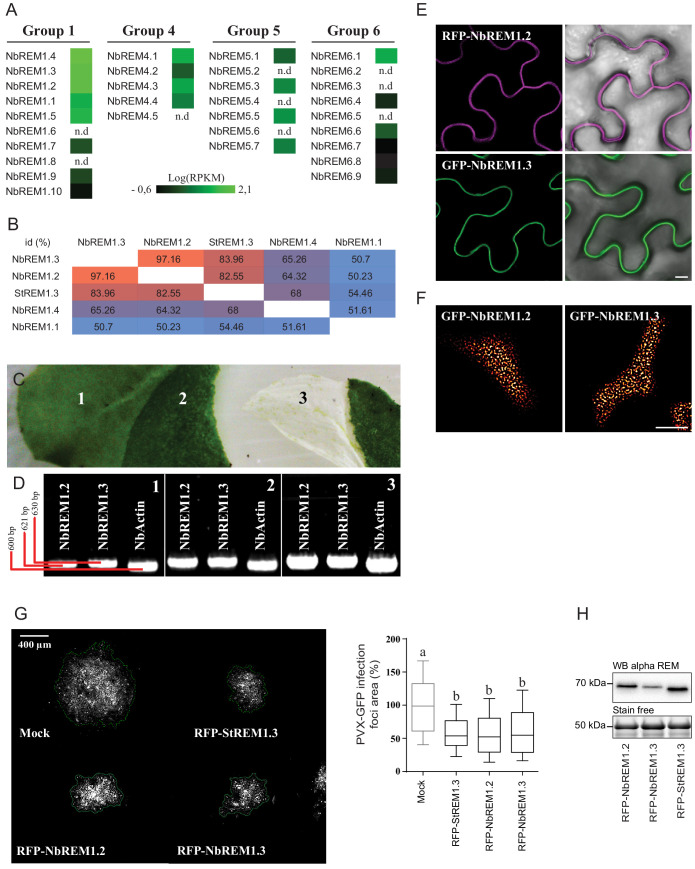


Original Figure 1 – figure supplement 2 panel G:

**Figure 4:**
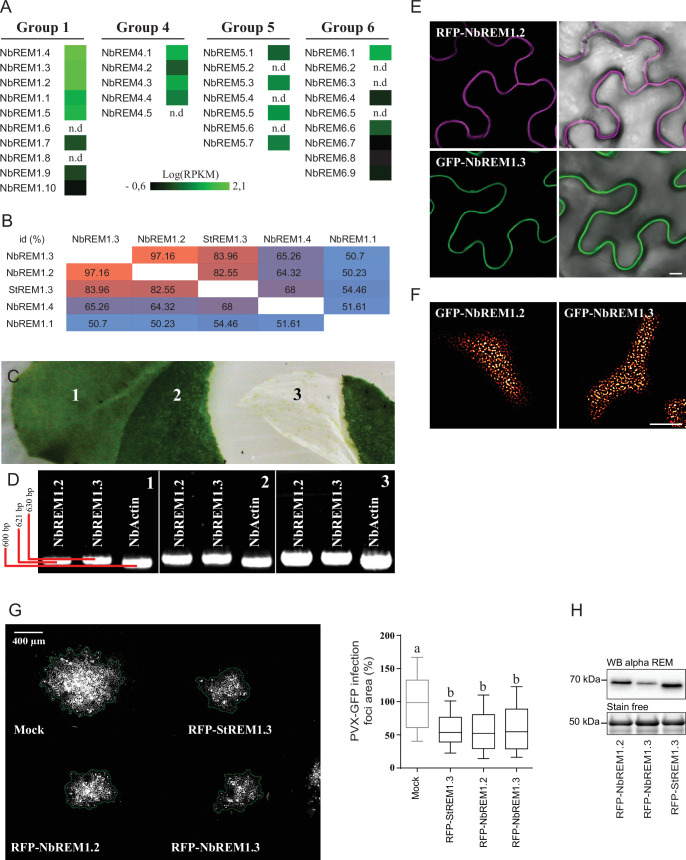


Corrected Figure 4 – figure supplement 1:

**Figure 5:**
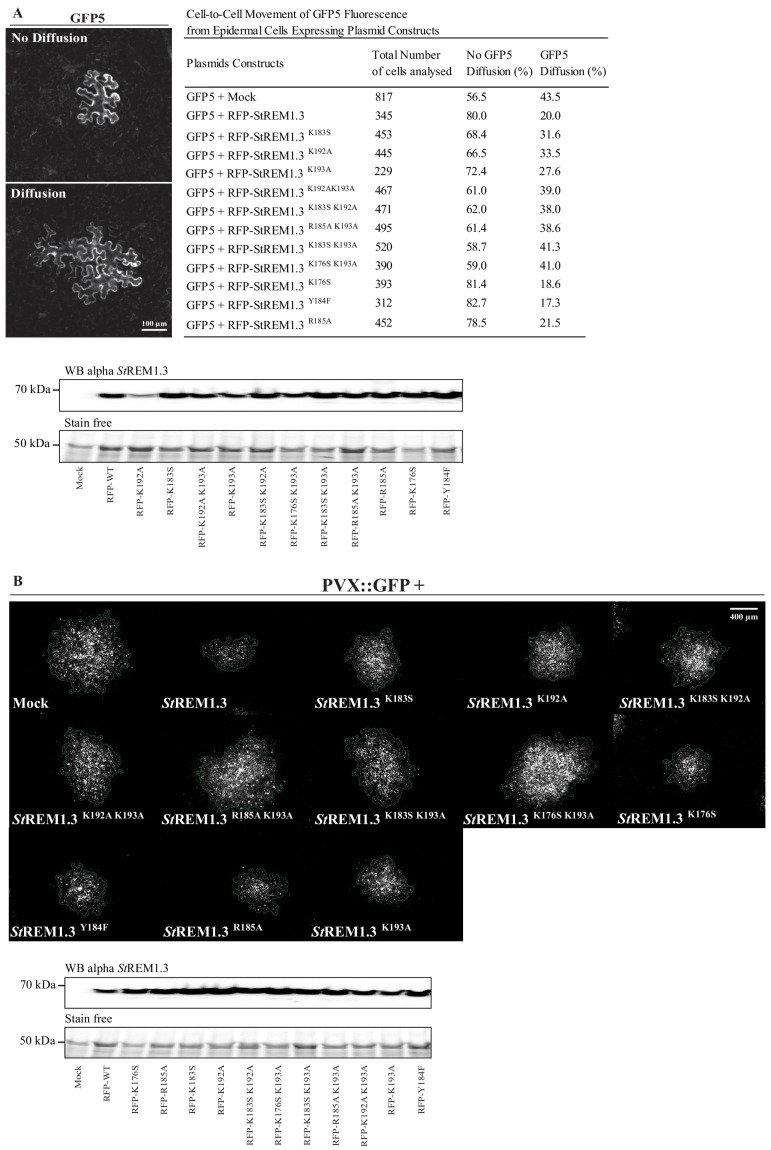


Original Figure 4 – figure supplement 1:

**Figure 6:**
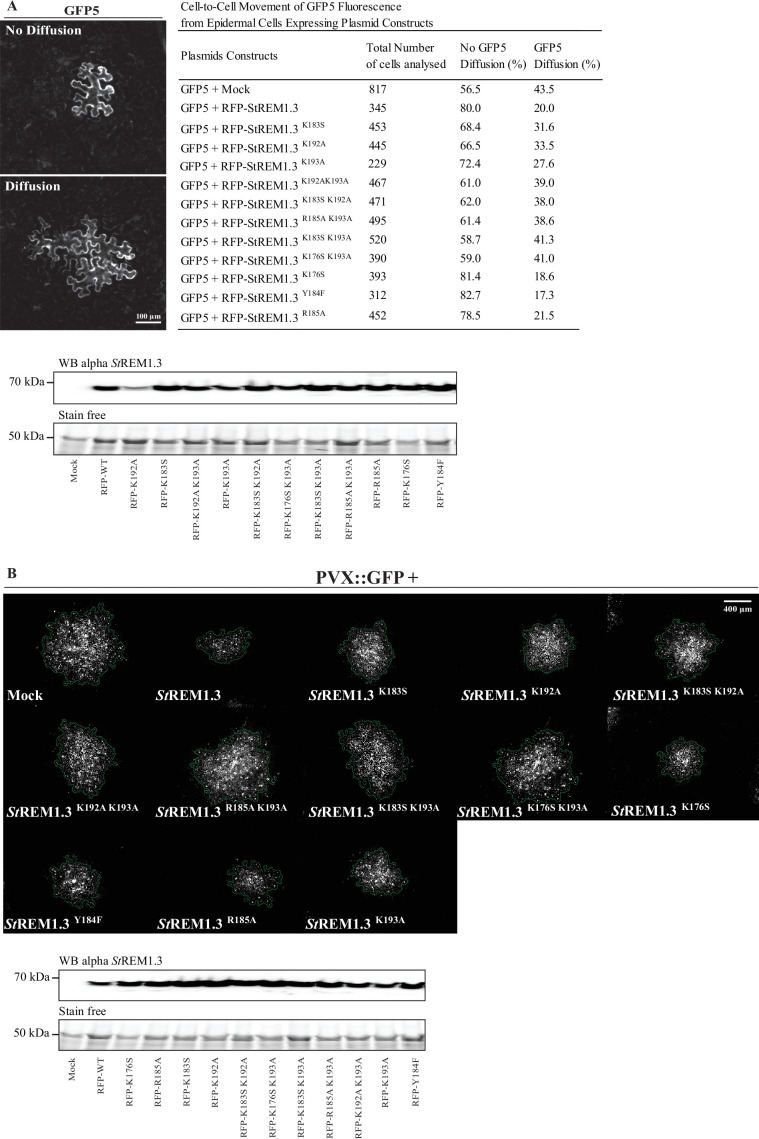


The article has been corrected accordingly.

